# Licoisoflavone A potentiates oxaliplatin efficacy in colorectal cancer by dual targeting arginine metabolism of tumor microenvironment and cancer cells

**DOI:** 10.1186/s13020-026-01428-7

**Published:** 2026-05-28

**Authors:** Lu Wang, Qing-rui Liu, Bing-wen Zhou, Chu-yue Huang, Ping-gang Ding, Yu-jing Dong, Pu-yang Gong, Zheng-xin Chen, Zhi-min Fan

**Affiliations:** 1 Jiangsu Clinical Innovation Center for Anorectal Diseases of T.C.M, Nanjing Hospital of Chinese Medicine Affiliated to Nanjing University of Chinese Medicine, Nanjing, 210022 China; 2 College of Pharmacy, Southwest Minzu University, Chengdu, 610041 China

**Keywords:** Licoisoflavone A, Colorectal cancer, Arginine metabolism, Oxaliplatin, Organoid

## Abstract

**Background:**

Colorectal cancer (CRC) remains a leading cause of cancer-related mortality, with resistance to oxaliplatin (Oxa) and its associated side effects posing major therapeutic challenges. Natural flavonoids have shown potential in the prevention and treatment of CRC.

**Purpose:**

This study aimed to investigate whether combining Oxa with the bioactive flavonoid licoisoflavone A (LA) derived from Glycyrrhiza species could exert enhanced antitumor effects against CRC and to explore the underlying mechanisms.

**Methods:**

A CT26 tumor-bearing mouse model was established to evaluate the therapeutic efficacy of Oxa in combination with LA in vivo. Mass spectrometry-based proteomics and metabolomics analyses were performed to identify differentially expressed proteins and metabolites between the Oxa group and the Oxa plus LA group. Differential expressed proteins and associated pathways were subsequently validated in HCT116 cells, SW480 cells, and patient-derived organoids. Furthermore, two complex organoid models—an organoid–lymphocyte co-culture system and an air–liquid interface organoid system—were developed to investigate the underlying mechanisms. Flow cytometry and Western blot analyses were employed in both in vitro and in vivo settings.

**Results:**

The combination of LA and Oxa significantly inhibited tumor growth in CT26 tumor-bearing mice. Arginine metabolism was significantly reduced following LA treatment. Arginase-1 (ARG1) was identified as a potential target of LA. LA significantly downregulated ARG1 expression, which was associated with an increased proportion of CD8⁺ T cells and reduced expression of CD69⁺ and PD-1, suggesting a potential alleviation of T cell exhaustion-related features. Moreover, LA inhibited CRC cell proliferation by suppressing ARG1-mediated activation of the PI3K/Akt/mTOR signaling pathway.

**Conclusions:**

LA is a natural flavonoid that targets arginine metabolism and enhances the therapeutic efficacy of Oxa in CRC treatment.

**Graphical abstract:**

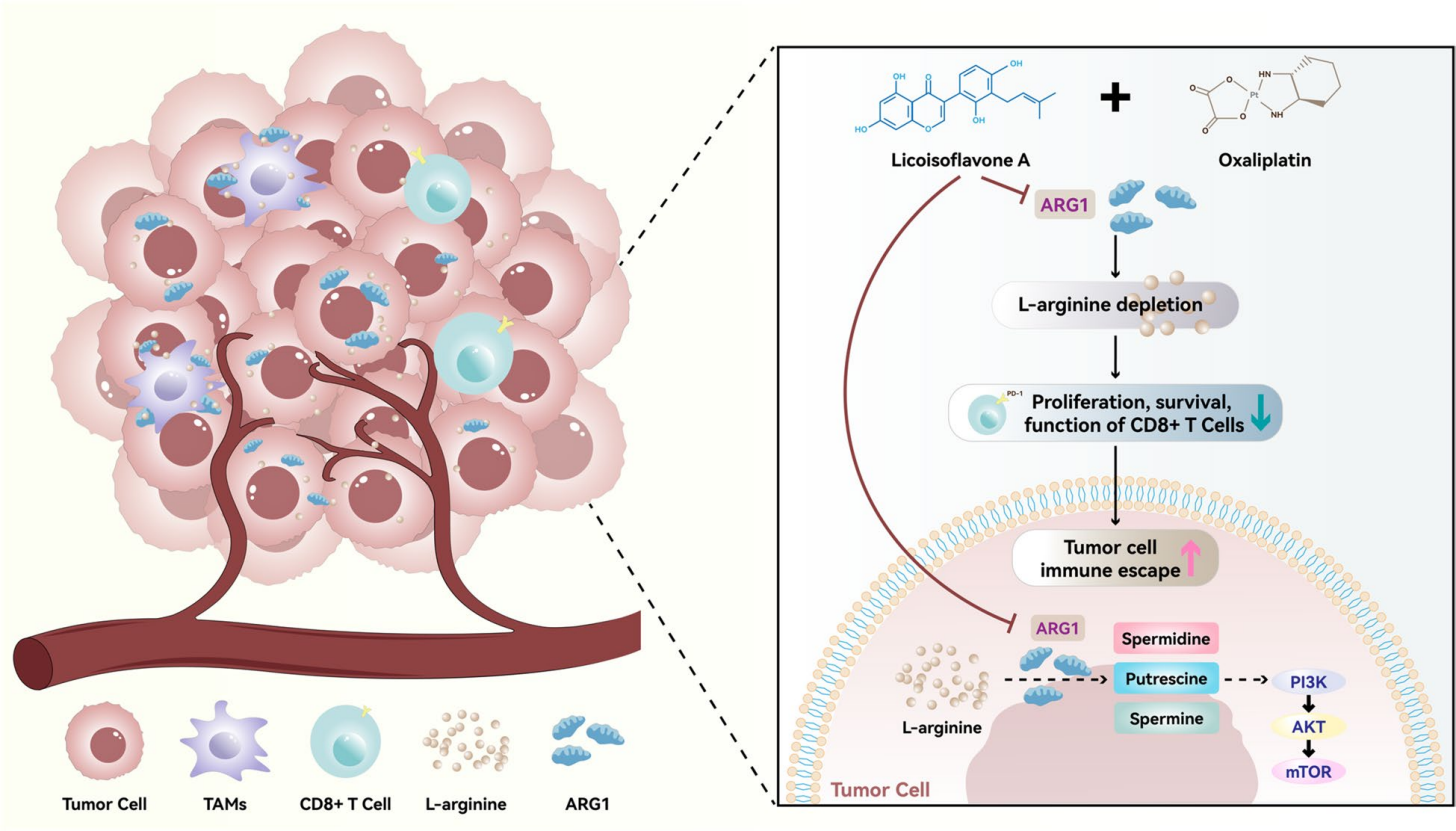

## Introduction

Colorectal cancer (CRC) remains a major global health concern, with increasing incidence and mortality rates. According to GLOBOCAN 2023, CRC is the third most commonly diagnosed cancer and the second leading cause of cancer-related death worldwide [1, 2]. Chemotherapy resistance and adverse side effects continue to pose significant challenges to achieving effective therapeutic outcomes. Oxaliplatin (Oxa), a third-generation platinum-based chemotherapeutic agent, has become a cornerstone in the treatment of advanced CRC. However, the development of resistance to Oxa is almost inevitable in most patients, ultimately leading to treatment failure and disease progression. As a result, the 5-year survival rate for patients with advanced CRC remains low and has shown limited improvement in recent years. In addition, Oxa has been reported to induce elevated levels of oxidative stress in cells [3], which may lead to cellular dysfunction and unpredictable cytotoxic effects. Strategies to enhance the drug’s efficacy while minimizing its side effects are currently under active investigation.

Traditional Chinese Medicine (TCM) has shown potential in the treatment of colorectal cancer (CRC), including the induction of tumor cell apoptosis and the inhibition of tumor cell proliferation [4–6]. TCM can be administered alone or in combination with chemotherapeutic agents for the treatment of advanced CRC and has been widely used during postoperative recovery and in conjunction with radiochemotherapy [7]. As an adjuvant therapy, TCM helps reduce the side effects of anticancer agents and enhances the efficacy of chemotherapy [8, 9]. Medicinal plants contain a wide variety of bioactive phytochemicals, including flavonoids, polyphenolic compounds, and saponins [10]. Flavonoids, in particular, are plant-derived bioactive compounds that have garnered significant attention in both nutrition and pharmacology due to their potent antioxidant, antitumor, anti-inflammatory, antibacterial, and antifungal properties [11]. Several studies have suggested the potential of flavonoids in CRC, including their antiproliferative effects, the ability to sensitize cancer cells to treatment, and a role in mitigating oxidative stress induced by pharmacological agents [12].

Licorice (the dried root of *Glycyrrhiza* species), a traditional herbal medicine classified as a “medicine food homology” substance, is rich in flavones with notable anticancer properties, including glycyrrhizin and licochalcone A [13–15]. These compounds exhibit promising antitumor potential through anti-inflammatory and anti-angiogenic mechanisms and significantly enhance the antitumor efficacy of Oxa in CRC models [16–18]. Isoflavones found in licorice, particularly licoisoflavone A (LA) and licoisoflavone B, are regarded as key bioactive constituents due to their well-defined structural features and diverse pharmacological activities. Our previous study showed that LA significantly suppressed colorectal cancer cell proliferation both in vitro and in vivo [19]. However, the comprehensive evaluation of the combined effects of LA and Oxa on CRC remains unexplored. Therefore, this study aimed to perform a systems-level analysis to investigate whether LA and Oxa exert enhanced antitumor effects in CRC and to explore the underlying mechanisms.

In the present study, the combined effects of LA and Oxa in suppressing CRC were confirmed in CRC cell lines, patient-derived organoids, and CT26 tumor-bearing mice. Subsequently, an integrated metabolomic and proteomic analyses were conducted to explore the potential mechanisms underlying the effects of LA, followed by further validation both in vivo and in vitro. Our findings suggest that LA may have potential as a chemosensitizer in CRC therapy.

## Material and methods

### Materials and reagents

Licoisoflavone A (purity > 98%, V88632) was purchased from Shanghai Yuanye Bio-Technology Co., Ltd. (Shanghai, China), dissolved in dimethyl sulfoxide (DMSO) to prepare a 6 mM stock solution, and stored at − 20 °C. Oxaliplatin (0102022) was obtained from Sichuan Meida Kangjiale Pharmaceutical Co., Ltd. (Chengdu, China). The arginase inhibitor Nω-hydroxy-nor-arginine (nor-NOHA, HY-112885) was purchased from MedChemExpress (Shanghai, China). The Cell Counting Kit-8 (CCK-8, E-CK-A362) was obtained from Elabscience (Wuhan, China). Trypsin (0.25%, KGA1519-5), phosphate-buffered saline (PBS, KGC3121-500) and DMSO (KGL2329-100) were purchased from KeyGEN BioTECH (Nanjing, China). McCoy’s 5 A medium (MD0900) was obtained from Qida Biotechnology (Shanghai, China). Dulbecco's modified Eagle's medium (DMEM, 11,965,092), Roswell Park Memorial Institute (RPMI) 1640 medium (11,875,093), and fetal bovine serum (FBS, A5256701) were purchased from Gibco (Grand Island, NY, USA). The 10% SDS-PAGE gel kit (PG610) was obtained from Epizyme Biomedical (Shanghai, China). The ECL Plus CLIA Test Kit (P00185M), SDS-PAGE buffer (5 ×, P0015L), cell lysis buffer (P0013), BCA protein assay kit (P0011) and penicillin–streptomycin (C0222) were purchased from Beyotime (Shanghai, China). Antibodies against GAPDH (60,004–1-Ig), phosphoinositide 3-kinase (PI3K, 60,225–1-Ig), Arginase-2 (ARG2, 14,825–1-AP), nitric oxide synthase 2 (NOS2, 22,226–1-AP) and mammalian target of rapamycin (mTOR, 66,888–1-Ig) were purchased from Proteintech Group (Wuhan, China). Antibodies against ARG1 (93,668), phosphorylated PI3K (p-PI3K, 4228), protein kinase B (Akt, 4691), and phosphorylated mTOR (p-mTOR, 5536) were obtained from Cell Signaling Technology (Danvers, MA, USA). phosphorylated protein kinase B (p-Akt) antibody (ab81283) was purchased from Abcam (London, UK). Antibodies against CD3 (317,319), CD8 (344,720), CD69 (310,924) and PD-1 (329,933) for flow cytometry analysis were obtained from BioLegend (Beijing, China). The Colorectal Cancer Organoid Kit (K2103-CR) was purchased from bioGenous (Shanghai, China). Matrigel (354234) was obtained from Corning (Corning, NY, USA). Collagenase IV (C4-28) and hyaluronidase (HX0514-1) were purchased from Sigma (St. Louis, MO, USA). The CellTiter-Glo Luminescent Cell Viability Assay Kit (G7570) was obtained from Promega (Madison, WI, USA). The ARG1 overexpression plasmid (G128858) was purchased from YouBio (Guangzhou, China).

### In vivo tumor model

Six-week-old male specific pathogen-free (SPF) BALB/c mice were housed in a temperature-controlled (21–23 °C) and humidity-controlled (45–65%) animal facility under a 12-h light/dark cycle, with free access to food and water. The mice were purchased from Jiangsu Huachuang Xinnuo Pharmaceutical Technology Co., Ltd. (license number: SCXK (Su) 2020–0009). All experimental procedures were conducted in accordance with the guidelines for the care and use of laboratory animals established by the National Institutes of Health.

CT26.WT cells in the logarithmic growth phase were harvested and washed twice with DPBS after removal of the culture medium. The cells were then subcutaneously inoculated into the right axillary region of BALB/c mice at a dose of 1 × 10⁶ cells in 100 μL per mouse. Cell viability was assessed prior to implantation. All procedures were performed under aseptic conditions. When the tumor volume reached approximately 100 mm^3^ (on day 7 post-inoculation), treatment was initiated. Mice received intraperitoneal injection of Oxa alone or Oxa in combination with oral administration of LA. Treatment continued for 2 weeks, during which the body weight of each mouse was monitored 2–3 times per week. One day after the final treatment, the mice were anesthetized and euthanized. Subcutaneous tumors were excised, photographed, weighed, and recorded. Tumor volume was measured every two days and calculated using the formula: (short diameter)^2^ × (long diameter)/2. Tumor tissues were divided into two portions: one was immediately fixed in 4% paraformaldehyde for hematoxylin and eosin (H&E) staining, while the other was snap-frozen in liquid nitrogen and stored at − 80 °C for subsequent metabolomics, proteomics, and Western blot analyses.

### Cell lines and cell culture

Human CRC cell lines HCT116 (RRID: CVCL_0291) and SW480 (RRID: CVCL_0546) were obtained from the Shanghai Institute of Cell Biology, Chinese Academy of Sciences (Shanghai, China). After thawing, HCT116 cells were cultured in McCoy’s 5 A medium supplemented with 15% fetal bovine serum (FBS), while SW480 cells were maintained in DMEM supplemented with 10% FBS. Mycoplasma detection was performed routinely before using the cells. All culture media contained 100 U/mL penicillin and 0.1 mg/mL streptomycin. Cells were incubated at 37 °C in a humidified atmosphere containing 5% CO₂. Cells were passaged at a ratio of 1:3 when confluence reached approximately 80%.

Murine colon cancer CT26.WT cells (RRID: CVCL_7256) were purchased from the Shanghai Institute of Cell Biology, Chinese Academy of Sciences (Shanghai, China) and cultured in RPMI-1640 medium supplemented with 10% FBS, 100 U/mL penicillin, and 0.1 mg/mL streptomycin. Mycoplasma detection was performed routinely before using the cells. Cells were passaged at a 1:3 ratio upon reaching over 80% confluence.

### Cell viability assay

Cell viability for both proliferation and cytotoxicity assays was assessed using the Cell Counting Kit-8 (CCK-8) according to the manufacturer's instructions. Briefly, following drug treatment, 10 μL of CCK-8 solution was added to each well, and the plates were incubated for 4 h in a humidified incubator. Absorbance was measured at 450 nm using a microplate reader (BioTek Synergy H1, VT, USA).

### Patient-derived colorectal cancer organoid culture

For classical three-dimensional (3D) culture, patient-derived colorectal cancer organoids were thawed from cryopreserved organoid lines. Organoids were resuspended in Matrigel, seeded into 6-well plates, and cultured using the Colorectal Cancer Organoid Kit. The culture medium was refreshed every 48 h. Organoids were passaged every 5–7 days either by mechanical dissociation using a pipette tip or, when necessary, with 1–2 mL of TrypLE^™^ Express (Gibco, NY, USA).

For air–liquid interface (ALI) culture, transwell inserts (PICM03050, Millicell-CM, Millipore) were placed into 12-well culture plates. Collagen solution was prepared on ice, and 0.25 mL of reconstituted collagen was added to each insert, followed by incubation at 37 °C to allow gelation. Tumor resection specimens were transferred into ice-cold PBS containing 1% penicillin–streptomycin and washed thoroughly to minimize bacterial contamination. Samples were then dissected into 2–3 mm pieces and further minced into fine fragments of approximately 0.1 mm. The fragments were collected in ice-cold Advanced DMEM/F-12 medium supplemented with 1% penicillin–streptomycin, washed thoroughly, and centrifuged at 400 × *g* for 5 min. The pellet was resuspended in 0.25 mL of ice-cold collagen solution, mixed thoroughly to ensure even distribution within the matrix, and plated onto the pre-solidified bottom collagen layer. The matrix was allowed to solidify at 37 °C for 30 min. Finally, 1 mL of CRC organoid culture medium was added to each well.

### Organoid-lymphocyte co-culture

T cell culture medium was prepared by supplementing RPMI-1640 with 10% FBS, 2 mM GlutaMAX, 1% penicillin–streptomycin, and 100 U/mL interleukin-2. Peripheral blood mononuclear cells (PBMCs) were thawed and cultured for 4 days in this medium in ultra-low attachment 6-well plates. Prior to co-culture, CRC organoids were dissociated into single cells. PBMCs and tumor cells were then co-cultured at a 1:1 ratio in ultra-low attachment 12-well plates at a total density of 1 × 10^5^ cells per well. Fresh medium was replenished every two days throughout the co-culture period.

### Organoids viability assay

Organoids were enzymatically dissociated into single cells and seeded into 384-well plates at a density of 500 cells per well. After 24 h, varying concentrations of drugs were added to each treatment group, while medium alone was used as the control. Cell viability was assessed 3 days after treatment using the CellTiter-Glo^®^ Luminescent Cell Viability Assay (CTG) (Promega Corporation, WI, USA), according to the manufacturer’s instructions.

### Organoid fluorescence staining

Organoid viability was assessed using a Calcein-AM and Propidium Iodide (PI) double-staining kit. Organoids were first washed with PBS to remove residual medium, then incubated with 500 μL of staining solution at 37 °C for 1 h in the dark. After incubation, organoids were immediately imaged using a high-content intelligent imaging analyzer (AvatarInsight, Suzhou, China). Viable cells (green fluorescence) and dead cells (red fluorescence) were simultaneously visualized under an excitation wavelength of 490 ± 10 nm.

### Metabolomics analysis

Serum metabolites were extracted by protein precipitation. Briefly, 100 μL of serum was mixed with four volumes of cold ethanol: methanol (1:1, v/v), vortexed, sonicated in an ice bath for 10 min, and kept at − 20 °C for 1 h. After centrifugation (12,000 rpm, 15 min, 4 °C), the supernatant was dried under vacuum and reconstituted in 100 μL ethanol: water (1:1, v/v). The clear supernatant was used for LC–MS analysis. Untargeted metabolomics was performed on a SHIMADZU LC-40 UHPLC coupled with a SCIEX TripleTOF 7600 system using a Waters ACQUITY UPLC HSS T3 column (2.1 × 100 mm, 1.8 μm). The mobile phases were 0.1% formic acid in water (A) and acetonitrile (B). The flow rate was 0.4 mL/min with a 5 μL injection, and the gradient was: 0 min 5% B, 2 min 5% B, 10 min 60% B, 15 min 70% B, 20 min 80% B, 22 min 95% B, 28 min 60% B, 32 min 30% B. Mass spectrometry was operated in both positive and negative ion modes (± 5500 V, ± 80 V declustering potential, ± 10 V collision energy, 500 °C, ion source gas 1/2: 50 psi, curtain gas 35 psi; scan m/z 100–1500). Raw data were converted to ABF format and processed in MS-DIAL for peak detection, deconvolution, alignment, and metabolite identification using MS/MS libraries (MassBank, HMDB, GNPS). Metabolites with |Log2FC|> 1, VIP > 1 and p < 0.05 were considered significantly different and subjected to KEGG pathway enrichment analysis using clusterProfiler.

### Proteomics analysis

Protein extraction and digestion were performed following the filter-aided sample preparation (FASP) protocol. Briefly, tissue samples were lysed in 4% SDS, 100 mM Tris–HCl (pH 7.6) with protease inhibitors, sonicated, and centrifuged at 12,000 rpm for 20 min at 4 °C. Protein concentration was determined using the BCA assay. Equal amounts of protein were reduced with 100 mM DTT (95 °C, 5 min), alkylated with 100 mM IAA (dark, 30 min), and digested with trypsin (1:50, w/w) at 37 °C overnight. Peptides were desalted using C18 spin columns and dried under vacuum. LC–MS/MS analysis was performed using a Vanquish Neo UHPLC coupled to an Orbitrap Astral mass spectrometer (Thermo Fisher Scientific). Peptides were separated on a C18 EASY-Spray column (2 µm, 150 µm × 15 cm) at 55 °C with a linear gradient of 4–99% acetonitrile (0.1% formic acid). Data-independent acquisition (DIA) was applied with 299 windows (2 m/z width), MS1 resolution 240,000, and HCD collision energy 25 eV. Raw data were analyzed using DIA-NN (v1.8.1) against the UniProt Mus musculus database (FDR ≤ 1%). Protein intensities were normalized and filtered (> 50% valid values), missing values imputed via KNN. Differential proteins were identified by fold change > 1.5 or < 0.67 and p < 0.05, and subjected to GO and KEGG enrichment analyses using clusterProfiler.

### Bioinformatic analysis

Integrated pathway analysis was conducted to identify shared KEGG pathways between proteomic and metabolomic datasets. The KEGG enrichment results from both omics were imported into R, and overlapping pathways were determined using the VennDiagram package. Pathways common to both datasets were considered as potential key metabolic processes linking protein expression and metabolite alterations. To ensure biological relevance, only pathways with p < 0.05 in both analyses were retained for further interpretation and visualization.

### Western blot analysis

Cells or organoids were treated with various concentrations of drugs and harvested for Western blot analysis. Total cellular proteins were extracted using a Western blot lysis buffer, and protein concentrations were determined using a BCA protein assay kit. Equal amounts of protein (20 μg per sample) were separated by 10% SDS-PAGE and transferred onto PVDF membranes. Membranes were blocked with 5% nonfat milk for 2 h and then incubated overnight at 4 °C with specific primary antibodies. After washing, membranes were incubated with appropriate secondary antibodies at the recommended dilutions for 1 h at room temperature. Immunoreactive bands were visualized using an imaging system (Tanon, Shanghai, China). Tumor tissue samples were processed using the same protocol.

### Flow cytometry analysis of tumor-infiltrating lymphocytes

Single-cell suspensions were prepared by digesting organoid–collagen structures with collagenase IV (300 U/mL) at 37 °C for 30 min, followed by one wash with 10 mL of Advanced DMEM/F-12. Cells were then resuspended in ice-cold FACS buffer (PBS containing 2% FBS) for subsequent antibody staining. For the co-culture system, tumor cells and PBMCs were directly harvested and washed once with FACS buffer. Cell staining was initiated with incubation in Zombie Aqua™ viability dye, followed by staining with a surface antibody cocktail containing anti-CD3, anti-CD8, anti-CD69, and anti-PD-1 antibodies. All incubations were performed at room temperature for 15–20 min in the dark. After staining, cells were washed with 1 mL of FACS buffer and centrifuged at 600 × g for 3 min. Annexin V (5 μL per 100 μL cell suspension) was then added and incubated at room temperature for 15–20 min in the dark before flow cytometric analysis using a BD FACSAria instrument.

For mouse tumor tissues, single-cell suspensions were prepared by enzymatic digestion of minced tumors in a buffer containing 2.5 mg/mL trypsin, 0.5 mg/mL collagenase IV, and 20 μg/mL DNase I at 37 °C for 30 min. The resulting suspension was filtered through a 40-μm nylon mesh and centrifuged at 400 × g for 5 min. The cell pellet was treated with red blood cell lysis buffer. Following lysis, cells were stained with Fixable Viability Stain 780 and Fc receptors were blocked using TruStain FcX™ PLUS (anti-mouse CD16/32) antibody. For immunophenotyping, cells were stained with the following fluorescently labeled antibodies: PE/Cyanine7 anti-mouse CD45, APC anti-mouse CD3, FITC anti-mouse CD8a, and PE anti-mouse CD279 (PD-1). Flow cytometry data were acquired using a Beckman CytoFLEX instrument and analyzed with FlowJo software (version 10).

### Biochemical analysis

Serum alanine aminotransferase (ALT) and aspartate aminotransferase (AST) levels were measured using the ALT assay kit (Jiancheng, Nanjing, China) and the AST assay kit. (Jiancheng, Nanjing, China).

### Immunohistochemistry (IHC) analysis

Ten pairs of human CRC tissues and the matched normal tissues were obtained from Nanjing Hospital of Chinese Medicine Affiliated to Nanjing University of Chinese Medicine. The specimens were fixed in 4% paraformaldehyde and embedded in paraffin. Briefly, paraffin-embedded tissue sections were deparaffinized in xylene and rehydrated through a graded ethanol series followed by distilled water. Antigen retrieval was performed by microwave boiling in 10 mM sodium citrate buffer (pH 6.0), after which sections were incubated with 3% hydrogen peroxide to block endogenous peroxidase activity. Tissue sections (5 μm thick) were incubated overnight at 4 °C with primary antibodies against ARG1 (1:400) and ARG2 (1:400). A horseradish peroxidase (HRP)-conjugated secondary antibody (1:1000) and DAB substrate were used for detection. Slides were counterstained with hematoxylin. Images were acquired using a microscope (Olympus CX-41, Japan), and quantitative IHC analysis was performed using ImageJ software.

### Cell transfection for gene overexpression

Gene overexpression via plasmid transfection is a commonly used method to study gene function. In this study, HCT116 or SW480 cells were seeded into 6-well plates at a density of 2 × 10^5^ cells/mL. After overnight incubation, ARG1 overexpression plasmid (G118829, YouBio, China) was added to designated wells, and transfection was carried out using Lipofectamine^™^ 2000 (11,668,027, Gibco, USA). After 48 h of incubation, cells with successful transfection were selected for further analysis. All cell lines were maintained in an incubator at 37 °C with 5% CO₂. The culture medium was replaced every two to three days.

### siRNA-mediated knockdown assay

Small interfering RNA targeting ARG1 (ARG1-siRNA, sense sequence: 5′-UGGAUUUGUACCAUUCUUCUG-3′) and negative control siRNA (Control-siRNA, sense sequence: 5′-UUCUCCGAACGUGUCACGUTT-3′) were designed and synthesized by GenScript (Nanjing, China). HCT116 and SW480 cells were seeded in six-well plates at a density of 2 × 10^5^ cells per well. Transfection was performed using Lipofectamine 2000 (11,668,019, Gibco, USA) following the manufacturer’s instructions. Cells were collected 24 h post-transfection for subsequent experiments.

### Surface plasmon resonance (SPR)

SPR analysis was performed using a Biacore T200 system (Cytiva, USA) at 25 °C. Recombinant human ARG1 or ARG2 protein (Novoprotein, China) was diluted to 10 μg/mL in sodium acetate buffer (pH 4.5) and immobilized onto a CM5 sensor chip via standard amine coupling chemistry. The immobilization levels were approximately 3000–4000 response units (RU). For binding analysis, LA was serially diluted in running buffer (HBS-EP +, 10 mM HEPES, 150 mM NaCl, 3 mM EDTA, 0.005% Tween-20, pH 7.4) and injected over the flow cells at a flow rate of 30 μL/min with an association time of 60 s and a dissociation time of 120 s. The sensor chip surface was regenerated between cycles using 10 mM glycine–HCl (pH 2.0). Binding data were processed by subtracting signals from a reference flow cell (without immobilized protein) and analyzed using Biacore Insight Evaluation Software (Cytiva, USA). Equilibrium dissociation constants (Kd) were calculated by fitting the data to a 1:1 Langmuir binding model.

### Cellular thermal shift assay (CETSA)-Western blot (WB)

CETSA-WB experiment was carried out as previously described [20]. Briefly, the soluble protein lysate of HCT116 cells was aliquoted into PCR tubes and treated with LA (20 μM) or DMSO for 1 h at room temperature prior to heat treatment for CETSA. The solutions were heated at the indicated temperatures (45–65 ℃) for 3 min, followed by cooling at 4 ℃ for 3 min in a thermocycler (Applied biosystems, USA). After centrifugation for 20 min (20,000 g, 4 ℃), the soluble supernatant was subjected to Western blotting.

### Statistical analysis

All analyses were performed using GraphPad Prism v10 (GraphPad Software, La Jolla, CA, USA). Data are presented as the mean ± SD. One-way analysis of variance (ANOVA) followed by Dunnett's test was used for multiple comparisons. P < 0.05 was considered statistically significant. All experiments were performed in at least three independent replicates.

## Results

### LA enhances the antitumor effect of Oxa in CT26-bearing mice

**Fig. 1:**
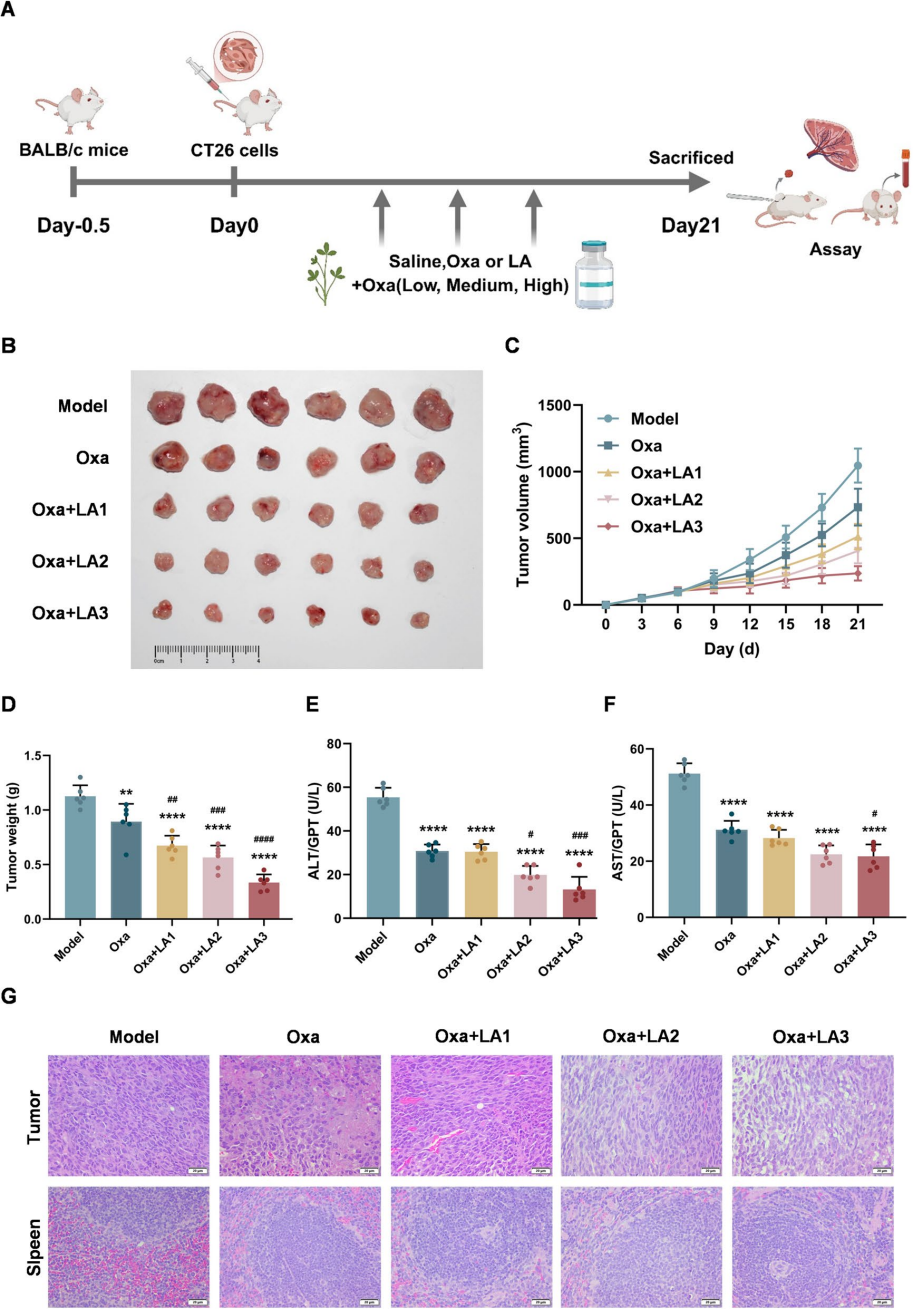
LA enhances the antitumor effect of Oxa in CT26 tumor-bearing mice. **A** Schematic diagram of the animal experiment design. **B** Image of the subcutaneous tumors in different groups of mice (n = 6). **C** The tumor volume curves of mice in different groups. **D** The weights of the tumors derived from CT26 tumor-bearing mice. **E–F** The plasma enzyme activities of ALT and AST. **G** Representative H&E staining of the tumor and spleen of mice (scale bar, 50 μm). Quantitative data were presented as mean ± SD. (n = 3). **p < 0.01, ****p < 0.0001 compared with Model. ^#^p < 0.05, ^##^p < 0.01, ^###^p < 0.001, ^####^p < 0.0001 compared with Oxa.

A CT26 tumor-bearing mouse model was used to evaluate the antitumor efficacy of LA and oxaliplatin (Oxa). BALB/c mice were subcutaneously inoculated with CT26.WT cells and, seven days post-injection, randomly assigned to one of five groups: the model group, the Oxa group (10 mg/kg), the low-dose LA (7.5 mg/kg) + Oxa (10 mg/kg) group, the middle-dose LA (15 mg/kg) + Oxa (10 mg/kg) group, and the high-dose LA (30 mg/kg) + Oxa (10 mg/kg) group (Fig. 1A). LA significantly enhanced the antitumor effect of Oxa in a dose-dependent manner, as reflected by reductions in tumor weight and volume compared with the model group (Fig. 1B–D). Oral administration of LA further enhanced the antitumor effect of Oxa, suggesting that the combination treatment suppressed tumor growth. To further evaluate the potential toxicity of LA, plasma ALT and AST levels were measured. No significant toxicity was observed (Fig. 1E, F). H&E staining showed increased necrosis, vacuolization, and disrupted tissue architecture in tumors from the middle- and high-dose LA + Oxa groups. Meanwhile, no notable toxicity was detected in the spleen (Fig. 1G). Collectively, these results suggest that LA enhances the antitumor efficacy of Oxa, and the combination did not appear to exacerbate Oxa-associated hepatotoxicity or splenic histopathological changes, indicating a favorable safety profile.

### Integrated proteomic and metabolomic analyses suggest alterations in arginine metabolism following LA treatment

**Fig. 2:**
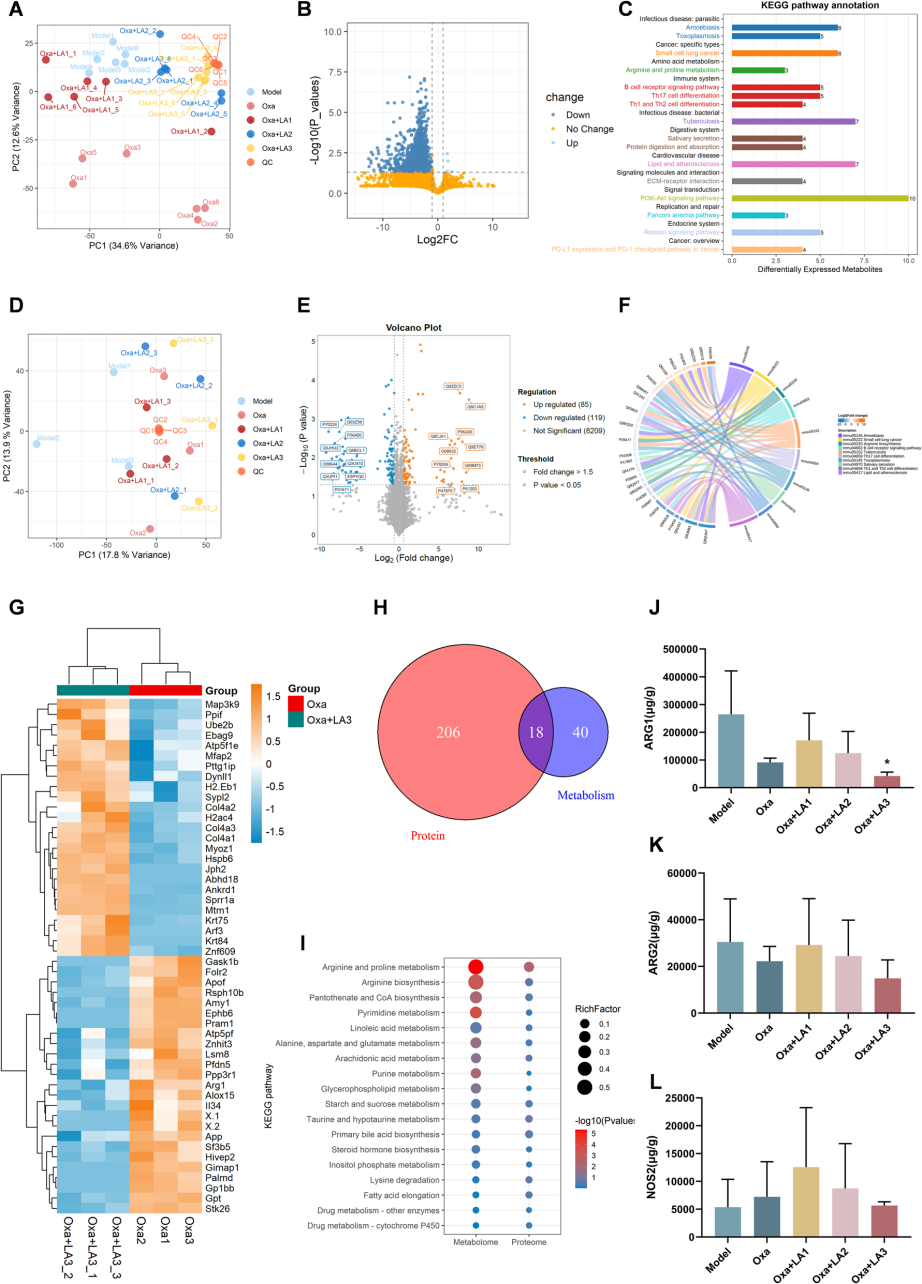
Integrated proteomic and metabolomic analyses suggest alterations in arginine metabolism following LA treatment. **A** The PCA score map of the metabolome showed obvious separation between the Model group, Oxa group, and Oxa combined with LA group. **B** Volcano plot of metabolites between Oxa and Oxa combined with LA3 groups. **C** KEGG enrichment analysis of differential metabolites. **D** The PCA score map of the protein group showed obvious separation between the Model group, Oxa group, and Oxa combined with LA3 groups. **E** Volcano plot of proteins between Oxa and Oxa combined with LA3 groups. **F** Chord diagram of KEGG pathways enriched by differential proteins. **G** Heatmap of representative differential proteins between Oxa and Oxa combined with LA3 groups. **H** Venn diagram between metabolome and proteome. **I** Bubble plot displaying joint enrichment results. **J–L** Serum expression levels of ARG1, ARG2 and NOS2. Quantitative data were presented as mean ± SD. (n = 3). *p < 0.05 compared with Model.

Metabolites and proteins, which represent key molecular components in biological systems, are increasingly analyzed using high-throughput approaches [21]. Proteomics and metabolomics, as powerful and effective postgenomic tools, have been widely applied in pharmacology research to elucidate the biomolecular mechanisms underlying TCMs. To investigate the metabolic basis associated with the enhanced antitumor response observed following Oxa and LA co-treatment, serum metabolomic profiling was performed among different groups. Principal component analysis (PCA) revealed clear group separation (Fig. 2A). Samples from the LA combined with Oxa group, especially those receiving higher LA doses, were distinctly segregated from the Oxa-alone and model groups. The tight clustering of QC samples indicated good analytical precision and reproducibility. Although all groups were profiled, subsequent differential analysis focused on the comparison between Oxa and Oxa combined with LA3 (high-dose), given that this group exhibited the most pronounced biological effect in previous pharmacodynamic experiments. Volcano-plot analysis identified a substantial number of significantly altered metabolites between the Oxa combined with LA3 group and Oxa group (Fig. 2B). Most differential metabolites showed a downward trend, suggesting that LA co-treatment was asscociated with broad metabolic changes. KEGG pathway enrichment demonstrated that these differential metabolites were mainly involved in amino acid metabolism, arginine and proline metabolism, PI3K–Akt signaling, ECM–receptor interaction, and protein digestion and absorption (Fig. 2C). These pathways are functionally linked to tumor energy metabolism and signal transduction, suggesting that LA may enhance the effects of Oxa, potentially associated with alterations in amino acid metabolism and PI3K/Akt-related metabolic signaling.

To further elucidate the molecular mechanisms underlying the metabolic alterations observed in the serum metabolomic analysis, a quantitative proteomic study was conducted across the Oxa and Oxa combined with LA groups with different LA doses. PCA revealed clear clustering among groups (Fig. 2D). Samples from the Oxa combined with LA groups were well separated from the Oxa and model groups. The tight aggregation of QC samples confirmed the analytical reliability and reproducibility of the dataset. Volcano-plot analysis identified numerous significantly altered proteins between Oxa and Oxa combined with LA3 (Fig. 2E). A total of 85 proteins were significantly upregulated and 119 proteins were significantly downregulated in the Oxa combined with LA3 group compared with Oxa alone, suggesting extensive proteomic changes associated with LA co-treatment. Updated KEGG pathway mapping demonstrated that these differential proteins were mainly associated with B cell receptor signaling, Th1/Th2 cell differentiation, Toll-like receptor signaling, Amoebiasis, and Lipid and atherosclerosis pathways (Fig. 2F). These immune- and inflammation-related pathways suggest that Oxa combined with LA3 treatment may be associated with alterations in immune-metabolic signaling and inflammatory stress response at the proteomic level. Hierarchical clustering of the top differential proteins further confirmed distinct expression signatures between Oxa and Oxa combined with LA3 groups (Fig. 2G), reflecting distinct proteomic signatures associated with high-dose LA co-treatment.

To identify the key biological pathways commonly altered at both the metabolite and protein levels, we performed an integrated KEGG pathway analysis combining the serum metabolomic and proteomic datasets. As shown in the Venn diagram (Fig. 2H), a total of 18 pathways were shared between the metabolomic and proteomic analyses, while 40 and 206 pathways were unique to the metabolome and proteome, respectively. Further joint enrichment mapping revealed that the common pathways were predominantly associated with amino acid metabolism (Fig. 2I), among which the arginine and proline metabolism pathway was the most significantly enriched. To further examine the key findings from the integrated analysis, we analyzed the expression of arginine metabolism-related proteins, including ARG1, ARG2, and NOS2 based on the proteomics data (Fig. 2J–L). Compared with the Oxa group, ARG1 expression was significantly decreased in the Oxa combined with LA3 group, while ARG2 and NOS2 showed a downward trend. These results suggest that arginine metabolism may be an important pathway potentially regulated by LA, warranting further mechanism investigation.

### LA enhances the inhibitory effect of Oxa on cell proliferation and reduces the expression of ARG1, ARG2 and NOS2 in patient-derived CRC organoids and CRC cells

**Fig. 3:**
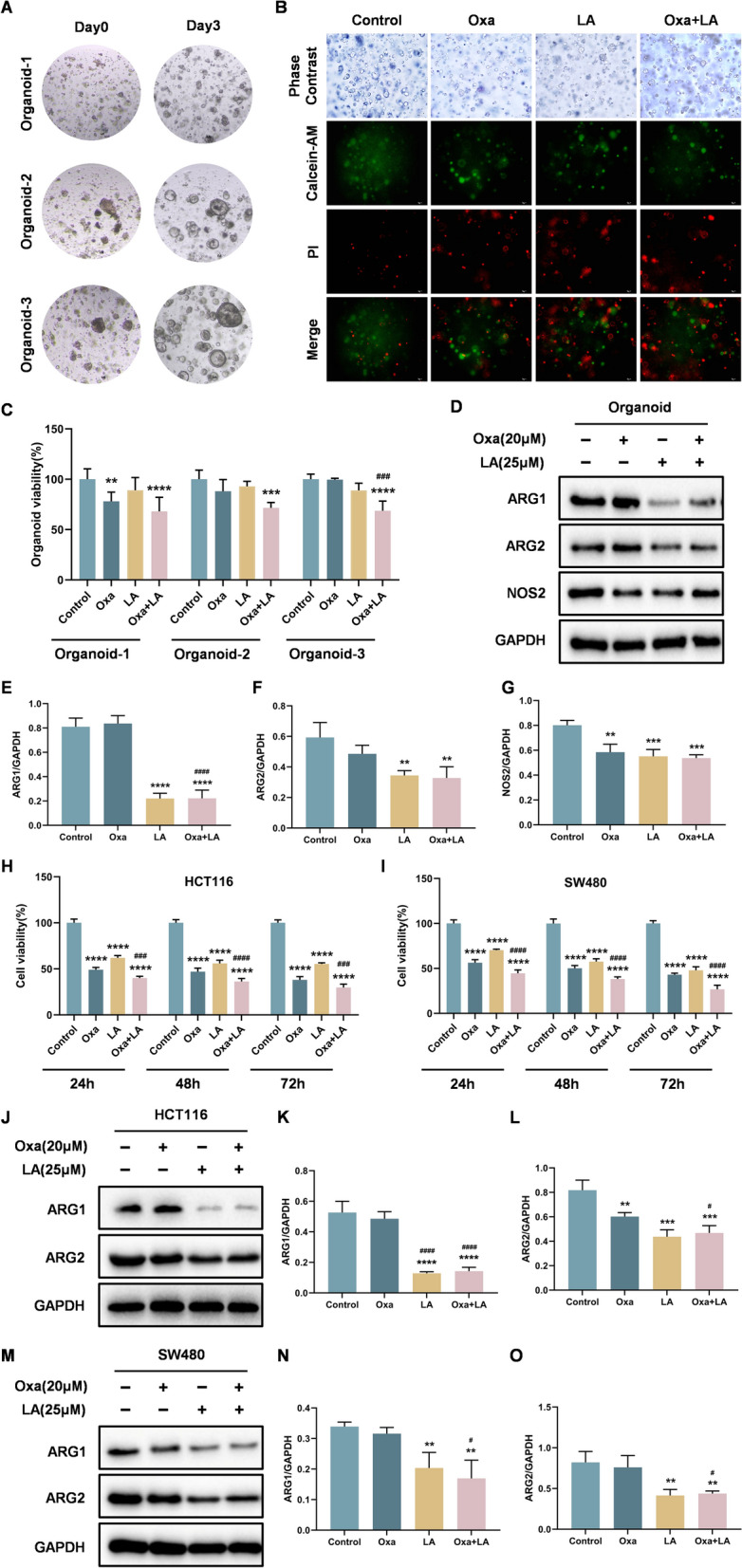
LA enhances the inhibitory effect of Oxa on cell proliferation and reduces the expression of arginine metabolism related proteins in patient-derived CRC organoids and CRC cells. **A** Representative images of three patient-derived CRC organoids. **B** Viability of organoid following treatment with various drugs was determined by fluorescence staining. **C** Viability of organoids following treatment with various drugs was measured by the CTG assay. **D** Western blot analysis of ARG1, ARG2 and NOS2 in organoids after treatment of different drugs. **E–G** Quantification of the band intensity of different proteins in organoids. **H-I** Viability of the two CRC cell lines (HCT116 and SW480 cells) following treatment with various drugs were measured by the CCK-8 assay. **J-L** Western blot analysis of ARG1, ARG2 and NOS2 in HCT116 cells following treatment with various drugs. **M–O** Western blot analysis of ARG1, ARG2 and NOS2 in SW480 cells following treatment with various drugs. Quantitative data were presented as mean ± SD. (n = 3). **p < 0.01, ***p < 0.001, ****p < 0.0001 compared with control. ^#^p < 0.05, ^###^p < 0.001, ^####^p < 0.0001 compared with Oxa.

Patient-derived tumor organoids are three-dimensional cultures established from individual patient tumors, with a high success rate of formation. These models can be expanded indefinitely and retain the morphological and genetic characteristics of the original tumors [22]. In this study, three patient-derived CRC organoids were cultured (Fig. 3A) to investigate the anti-tumor effects of LA, Oxa, and their combination. Treatment with LA combined with Oxa inhibited organoid viability more than either agent alone (Fig. 3B, C). In parallel, the expression of key arginine metabolism–related proteins, including ARG1, ARG2, and NOS2, was significantly downregulated upon combination treatment (Fig. 3D–G), with the reduction in ARG1 and ARG2 being more pronounced than that of NOS2. Notably, LA exerted a pronounced inhibitory effect on ARG1 expression. Compared with Oxa treatment alone, the combination of LA and Oxa resulted in a significantly greater suppression of ARG1. In contrast, no significant difference was observed in ARG2 inhibition. These findings suggest that LA may be associated with alterations in the arginine metabolism pathway, with a predominant effect on ARG1. To further validate these findings, the cytotoxic and inhibitory effects of LA, Oxa, and their combination were evaluated in CRC cell lines. HCT116 and SW480 cells were treated with LA, Oxa, or both drugs for 24, 48, and 72 h. Cell viability, measured by CCK-8 assay, showed that combination treatment significantly inhibited cell proliferation in a time-dependent manner (Fig. 3H, I). Consistent with organoid data, the effects of different treatments on ARG1 and ARG2 expression were assessed in HCT116 (Fig. 3J–L) and SW480 cells (Fig. 3M–O). The results showed that the combination of LA and Oxa significantly downregulated ARG1 and ARG2 expression in both cell lines. Collectively, these findings suggest that LA enhances the antitumor effect of Oxa by inhibiting arginase expression, highlighting a potential role of arginine metabolism in the observed effects.

### ARG1 is a potential target of LA in CRC treatment

**Fig. 4:**
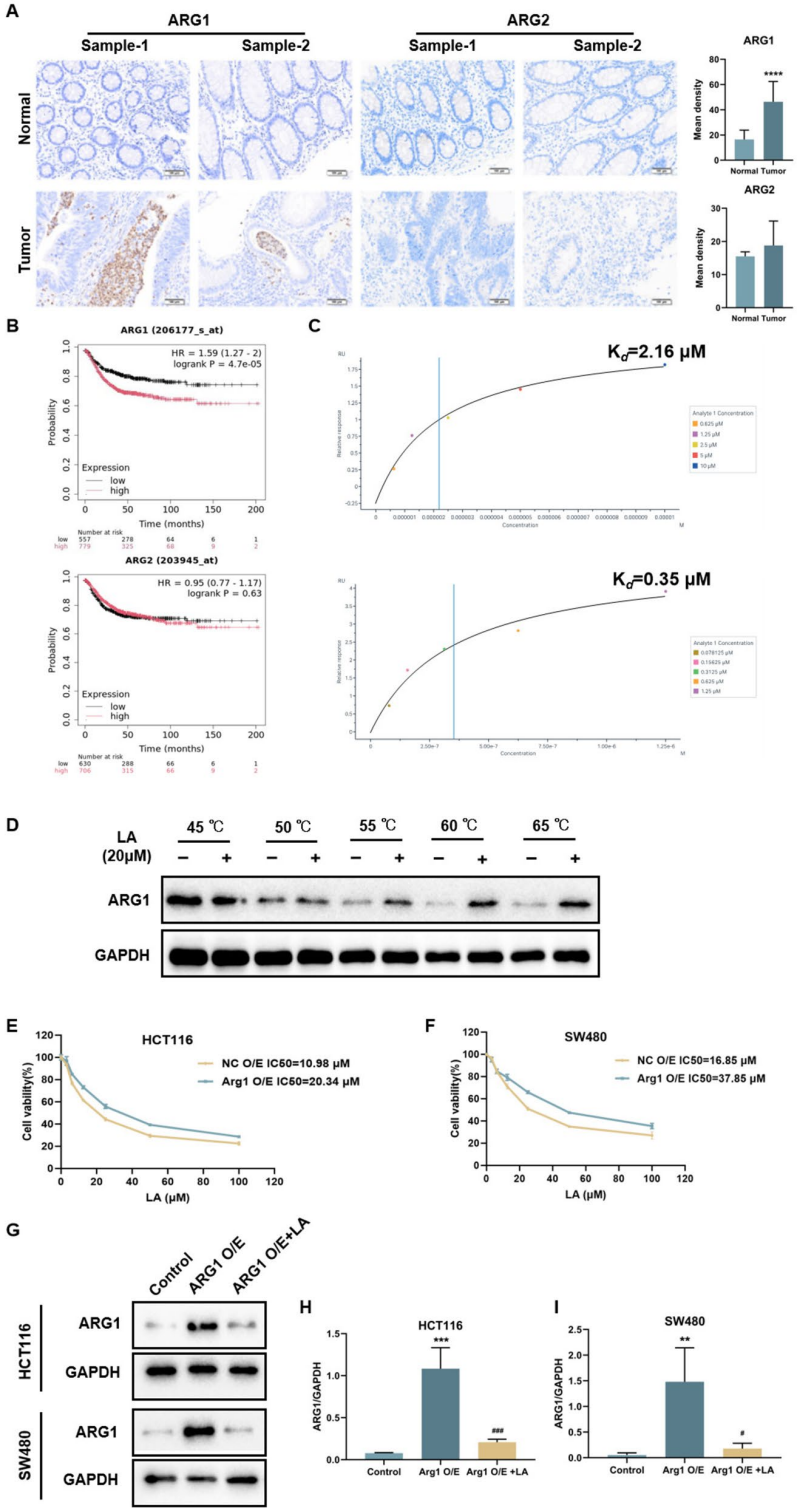
ARG1 is a potential target of LA in CRC treatment. **A** Representative images of IHC staining and quantification of ARG1 and ARG2 in clinical CRC tissues and adjacent noncancerous tissues (scale bar, 100 µm), (n = 10). **B** Survival curves of ARG1 and ARG2. **C** SPR assay of LA with recombinant human ARG1 and ARG2. **D** CETSA-WB experiment to further confirm the interaction between LA and ARG1 protein. **E, F** ARG1-overexpressing HCT116 and SW480 cells and vector control-transfected cells were exposed to LA for 24 h. Cell viability was measured by CCK-8 assay. **G–I** Western blot analysis of ARG1 in control cells, ARG1-overexpressing cells and ARG1-overexpressing cells treated with LA. Quantitative data were presented as mean ± SD. (n = 3). **p < 0.01, ***p < 0.001, ****p < 0.0001 compared with normal or control. ^#^p < 0.05, ^###^p < 0.001 compared with Oxa.

Previous studies have reported that ARG1 is highly expressed in CRC and facilitates tumor immune evasion, thereby promoting rapid tumor progression [23, 24]. However, few studies have investigated the role of ARG2 in CRC. To evaluate the expression levels of ARG1 and ARG2, IHC analysis was performed on clinical CRC tissue samples (n = 10) (Fig. 4A). The results showed that ARG1 expression was significantly higher in CRC tissues compared to adjacent noncancerous tissues, whereas ARG2 expression was low and showed no significant difference between tumor and normal tissues. Moreover, high ARG1 expression was associated with decreased survival probability, suggesting its potential as a prognostic marker in CRC. In contrast, ARG2 expression did not significantly correlate with patient survival (Fig. 4B), indicating a limited role for ARG2 in CRC pathogenesis. To explore the potential interaction between LA and arginase proteins, a surface plasmon resonance (SPR) assay was conducted. SPR analysis indicated that LA could bind recombinant human ARG1 and ARG2 proteins with Kd values of 2.16 μM and 0.35 μM, respectively (Fig. 4C). Although LA exhibited binding affinity for both ARG1 and ARG2, and showed stronger binding to ARG2 in SPR analysis, the functional relevance of ARG2 in CRC remains less established based on our clinical tissue expression and survival data. Therefore, ARG1 may play an important role in the effects of LA observed in this study, while the potential contribution of ARG2 cannot be excluded and warrants further investigation. To further substantiate that ARG1 is a functional target of LA, a cell lysate CETSA-WB experiment was carried out to support the direct interaction with LA. Protein extracts from HCT116 cells were treated with LA (20 μM) or DMSO, and subjected to CETSA heat pulse followed by soluble protein extraction. ARG1 displayed significant thermal stabilization in LA treatment group (Fig. 4D). All these findings suggest that LA may directly bind to ARG1.

To further examine the role of ARG1 in LA-mediated antitumor effects, HCT116 and SW480 cells were engineered to overexpress ARG1. Overexpression of ARG1 significantly increased the IC50 values of LA in both HCT116 (Fig. 4E) and SW480 (Fig. 4F) cells, indicating reduced sensitivity to LA-induced cytotoxicity. Notably, ARG1 expression in these overexpressing cells was significantly downregulated following LA treatment (Fig. 4G–I). Collectively, these findings provide strong evidence that ARG1 plays a critical role in regulating CRC cell proliferation and represents a key molecular target of LA.

### LA ameliorates T cell exhaustion by ARG inhibition in organoid-lymphocyte co-culture and air–liquid interface (ALI) organoids systems

High ARG1 expression may lead to depletion of essential amino acids, immunosuppression, and enhanced tumor progression [25]. Arginine and its metabolites possess strong immunomodulatory properties. Recent studies have shown that T cells residing in an arginine-rich microenvironment exhibit enhanced antitumor activity, which may be attributed to improved survival, metabolic reprogramming, and maintenance of a central memory-like phenotype [26].

**Fig. 5:**
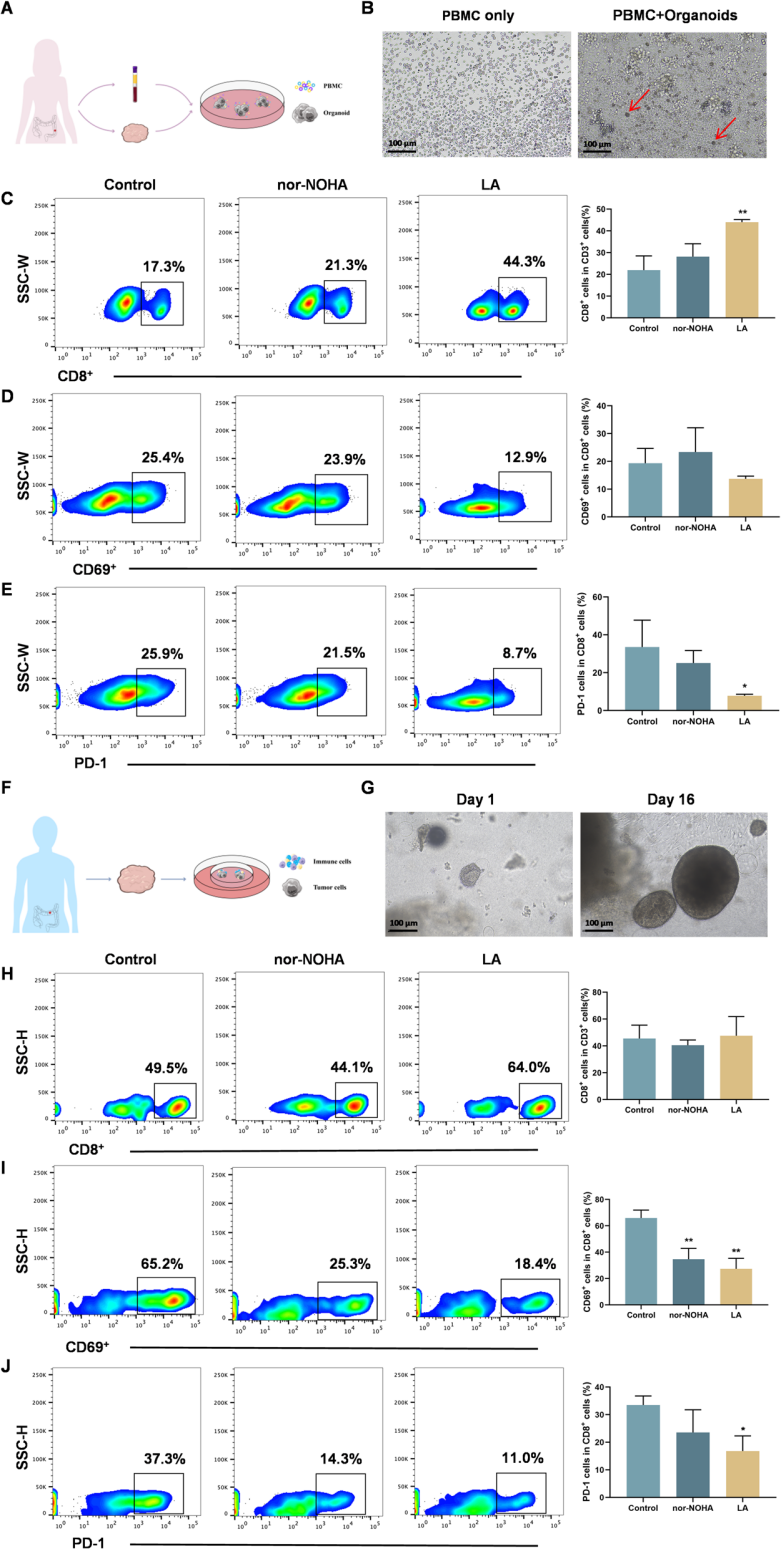
LA ameliorates T cell exhaustion by ARG inhibition in organoid-lymphocyte co-culture and ALI organoids systems. **A** Schematic diagram of organoid-lymphocyte co-culture system. **B** Representative images of organoid and PBMCs. **C-E** The representative images and quantitative analysis of CD8 + T cells, CD69^+^ and PD-1 in organoid-lymphocyte co-culture system after treatment with different drugs by flow cytometry assay. **F** Schematic diagram of air–liquid interface organoids culture system. **G** Representative images of air–liquid interface organoids. **H-J** The representative images and quantitative analysis of CD8 + T cells, CD69^+^ and PD-1 in air–liquid interface organoids after treatment with different drugs by flow cytometry assay. *p < 0.05, **p < 0.01 compared with control.

To investigate whether LA is associated with changes in T cell exhaustion-related features, potentially involving ARG1, two complex organoid models were established: an organoid–lymphocyte co-culture system and an ALI organoid system, both of which recapitulate key features of the tumor immune microenvironment (TIME) in vitro. In the organoid–lymphocyte co-culture system, CRC organoids were dissociated into single cells and co-cultured with PBMCs at a 1:1 ratio (Fig. 5A, B). Following treatment with LA or the arginase inhibitor nor-NOHA, tumor cells and PBMCs were collected, washed with FACS buffer, and analyzed by flow cytometry. LA treatment significantly increased the proportion of CD8⁺ T cells (Fig. 5C) and reduced the population of PD-1⁺ cells (Fig. 5E), while showing a trend toward decreased frequencies of CD69⁺ cells (Fig. 5D).

In the ALI organoid system, CRC organoids derived from primary tumor resections were directly cultured in ALI plates (Fig. 5F, G). Treatment with LA led to a modest increase in CD8⁺ T cells (Fig. 5H) and a significant reduction in the population of CD69⁺ (Fig. 5I) and PD-1⁺ (Fig. 5J) cells. Together, these findings suggest that LA is associated with changes in T cell exhaustion-related features in the TIME, potentially involving arginine metabolism.

### LA inhibits the PI3K/Akt/mTOR signaling pathway via ARG1 suppression in CRC cells and 3D organoids

**Fig. 6:**
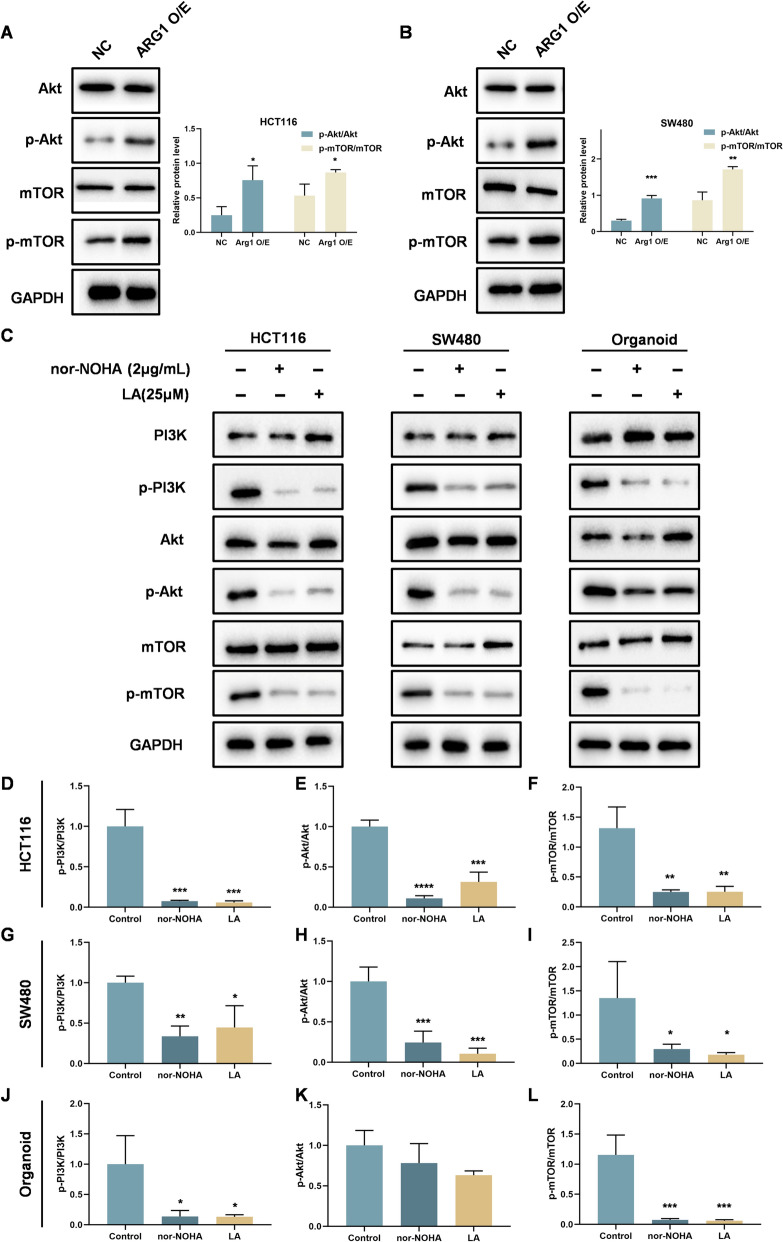
LA inhibits the activation of the PI3K/Akt/mTOR signaling pathway in CRC cells and 3D CRC organoids. **A** Western blot analysis of phosphorylation of Akt and mTOR in HCT116 cells with overexpression of Arg1. **B** Western blot analysis of phosphorylation of Akt and mTOR in SW480 cells with overexpression of Arg1. **C** Western blot analysis of p-PI3K, PI3K, p-Akt, Akt, p-mTOR and mTOR in CRC cells and organoids after treatment of different drugs. **D-L** Quantification of the band intensity of different proteins in CRC cells and organoids. Quantitative data were presented as mean ± SD. (n = 3). *p < 0.05, **p < 0.01, ***p < 0.001, ****p < 0.0001 compared with control.

Arginine can be converted into polyamines through the catalytic activity of arginase or agmatinase [27]. Elevated polyamine levels are often associated with poor tumor prognosis and contribute to tumor progression through multiple mechanisms. It is well established that abnormal activation of the PI3K/Akt/mTOR signaling pathway disrupts the regulation of cell proliferation and survival, ultimately promoting tumorigenesis, metastasis, and resistance to therapy [28]. Having confirmed that ARG1 overexpression enhances phosphorylation of Akt and mTOR in CRC cells (Fig. 6A, B), we next investigated whether LA exerts inhibitory effects on this signaling pathway. As shown in Fig. 6C, LA treatment significantly suppressed the levels of phosphorylated PI3K (p-PI3K), Akt (p-Akt), and mTOR (p-mTOR) in both HCT116 (Fig. 6D) and SW480 cells (Fig. 6E). Consistent with these cellular findings, a similar downregulation of pathway activity was observed in 3D CRC organoids following LA treatment (Fig. 6F).

**Fig. 7:**
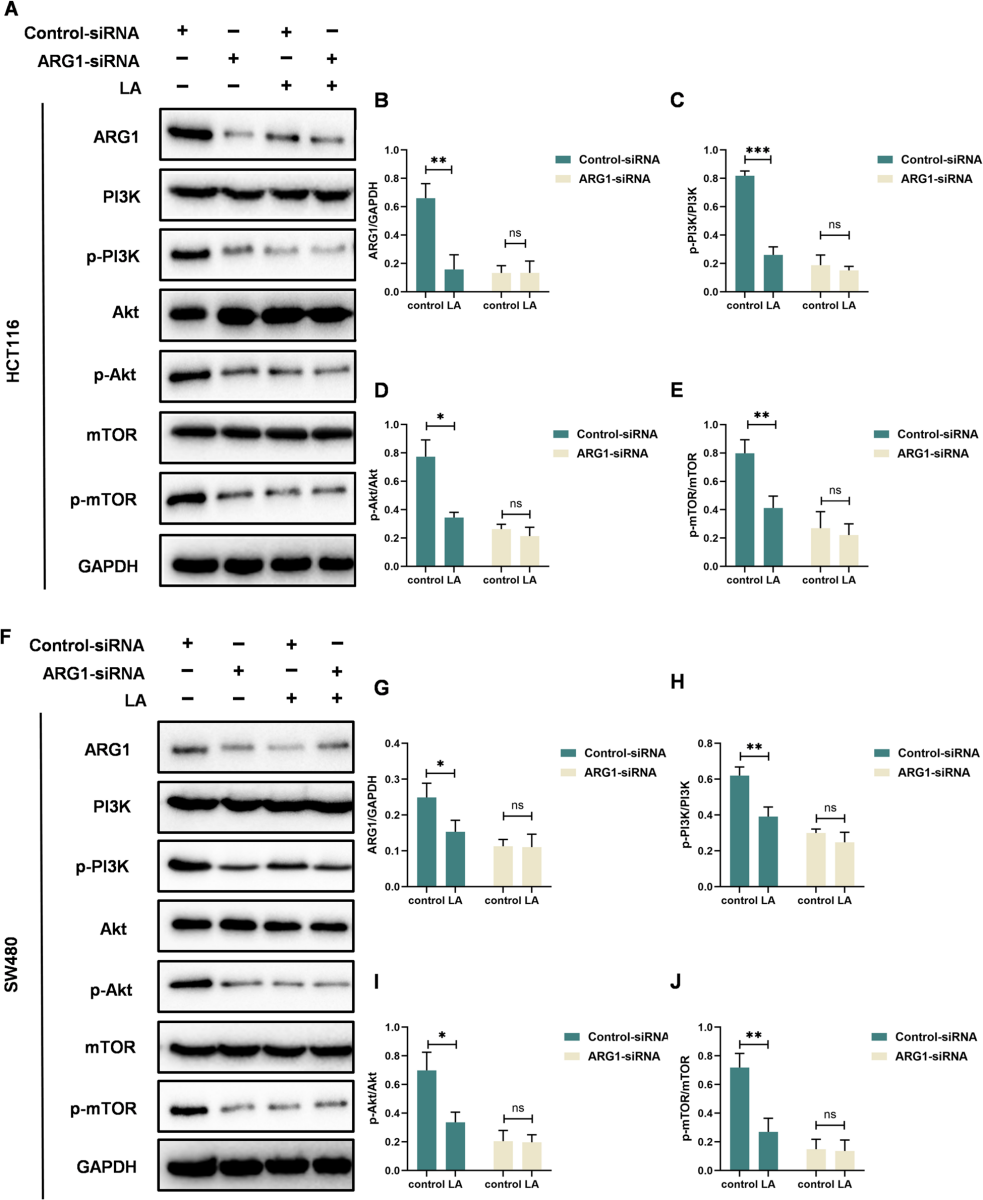
LA inhibits the activation of the PI3K/Akt/mTOR signaling pathway via ARG1 suppression in CRC cells. **A** Western blot analysis of ARG1 and the phosphorylation of PI3K/Akt/mTOR signaling pathway in HCT116 cells transfected with ARG1-siRNA after treatment with LA (25 μM). **B-E** Quantification of the band intensity of different proteins in HCT116 cells. **F** Western blot analysis of ARG1 and the phosphorylation of PI3K/Akt/mTOR signaling pathway in SW480 cells transfected with ARG1-siRNA after treatment with LA (25 μM). **G-J** Quantification of the band intensity of different proteins in SW480 cells. Quantitative data were presented as mean ± SD. (n = 3). *p < 0.05, **p < 0.01, ***p < 0.001. ns: not significant.

To further examine whether the effects of LA on the PI3K/Akt/mTOR signaling pathway via ARG1 suppression, we performed ARG1 knockdown using siRNA in HCT116 and SW480 cells. LA treatment failed to further suppress the phosphorylation of PI3K, Akt, or mTOR both in HCT116 cells (Figs. 7A–E) and SW480 cells (Figs. 7F–J). These findings suggest that ARG1 is required for LA-mediated inhibition of the PI3K/Akt/mTOR signaling pathway.

### LA suppresses colorectal tumor growth by dual targeting arginine metabolism of tumor microenvironment and cancer cells in vivo

**Fig. 8:**
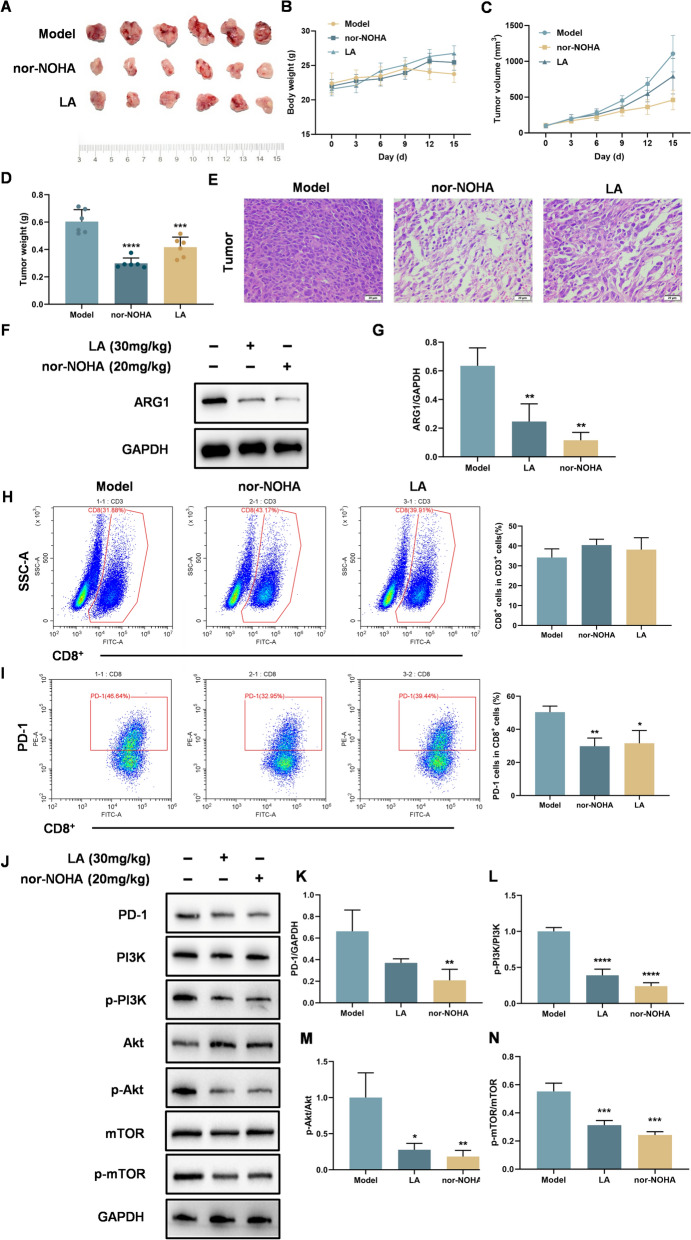
LA suppresses colorectal tumor growth by dual targeting arginine metabolism of tumor microenvironment and cancer cells in vivo. **A** Image of the subcutaneous tumors in different groups of mice (n = 6). **B** The weights of mice in different groups. **C** The tumor volume curves of mice in different groups. **D** The weights of the tumors derived from CT26 tumor-bearing mice. **E** Representative H&E staining of the tumor of mice. **F,G** Western blot analysis of ARG1 in tumor tissue of different groups. **H** The representative images and quantitative analysis of CD8 + T cells in tumor tissue after treatment with different drugs by flow cytometry assay. **I** The representative images and quantitative analysis of PD-1 in tumor tissue after treatment with different drugs by flow cytometry assay. **J** Western blot analysis of PD-1, p-PI3K, PI3K, p-Akt, Akt, p-mTOR and mTOR in tumor tissue after treatment with different drugs. **K-N** Quantification of the band intensity of different proteins in tumor tissue. Quantitative data were presented as mean ± SD. (n = 3). *p < 0.05, **p < 0.01, ***p < 0.001, ****p < 0.0001 compared with model.

To further examine whether the antitumor effects of LA are associated with ARG1, an in vivo CT26 tumor-bearing mouse model was established. Mice were randomly assigned to one of three groups: model control, nor-NOHA (20 mg/kg), and LA (30 mg/kg). In vivo evaluation showed that both LA and nor-NOHA downregulated ARG1 expression and significantly inhibited tumor growth without inducing notable toxicity, as evidenced by increased body weight and reduced tumor weight and volume compared to the model group (Fig. 8A–D). Histological analysis by H&E staining further supported the treatment efficacy. Tumors from LA- and nor-NOHA- treated mice exhibited extensive necrosis, vacuolization, and disrupted tissue architecture (Fig. 8E). Western blot analysis of subcutaneous tumors confirmed that LA treatment significantly reduced ARG1 expression levels (Fig. 8F, G), consistent with the in vitro findings. To evaluate the immune response within the tumor microenvironment, flow cytometry was performed on tumor-infiltrating lymphocytes. Both LA and nor-NOHA treatments showed a tendency to increase CD8⁺ T cell infiltration (Fig. 8H) and significantly reduced PD-1 expression on T cells (Fig. 8I), suggesting changes in T cell exhaustion-related features. Furthermore, Western blot analysis of tumor tissues demonstrated that LA downregulated PD-1 and suppressed the phosphorylation of PI3K, Akt, and mTOR in a dose-dependent manner (Fig. 8J–N), further suggesting its role in modulating the ARG1–PI3K/Akt/mTOR axis in vivo.

## Discussion

CRC is one of the most common malignancies worldwide and is characterized by a high rate of postoperative recurrence and metastasis, which remain major clinical challenges [29, 30]. Previous studies have reported that ARG1 overexpression significantly promotes metastatic colonization in colon cancer [31], and our findings further showed that ARG1 expression is markedly elevated in CRC tissues compared to adjacent normal tissues. In this study, we demonstrate for the first time that LA significantly enhances the antitumor efficacy of Oxa by targeting arginine metabolism, specifically through the inhibition of ARG1. LA-mediated downregulation of ARG1 not only suppressed CRC cell proliferation via the PI3K/Akt/mTOR signaling pathway but also alleviated T cell exhaustion within the TIME.

Licorice and its monomeric constituents exhibit a wide range of biological activities, including antioxidant, antitumor, antidepressant, anti-inflammatory, hepatoprotective, and spasmolytic effects [32]. Among these constituents, isoflavones are recognized as key bioactive compounds due to their well-defined structural features and diverse pharmacological properties. For example, licoisoflavone B has shown therapeutic potential for treating respiratory tract infections [33], while LA has been reported to inhibit pathological cardiac hypertrophy and alleviate acute lung injury [34]. However, studies investigating the antitumor efficacy of LA remain scarce. Although our previous work demonstrated that LA inhibits CRC progression by targeting the CDK2-related signaling pathway [19], its combined effects with Oxa and the underlying mechanisms had not yet been explored. The current study provides new insights into this combination strategy and suggests a potential therapeutic approach for CRC treatment.

ARG1, a key enzyme in the urea cycle, catalyzes the hydrolysis of arginine into urea and ornithine, thereby regulating the proliferation, differentiation, and function of various cell types [31]. ARG1 is widely expressed in tumors, and its elevated activity is strongly associated with advanced disease stages and poor clinical outcomes [35]. On one hand, ARG1 depletes arginine, an amino acid essential for T cell activation and proliferation [36]. Increasing arginine availability within the TIME has been shown to promote the expansion of tumor-infiltrating T cells, enhance antitumor immune responses, and improve the efficacy of immunotherapy. Given the central role of arginine metabolism in shaping TIME, pharmacological inhibition of ARG1, either as a monotherapy or in combination with immune checkpoint inhibitors (ICIs), has emerged as a promising strategy in cancer immunotherapy [37]. Moreover, plasma arginine levels may serve as a potential biomarker for guiding personalized therapies targeting the arginine metabolic pathway in conjunction with ICIs [38]. On the other hand, ARG1 also contributes to collagen synthesis and cellular bioenergetics, processes that are critical for malignant cell proliferation and invasiveness [39, 40]. ARG1 activation has been implicated in the metastatic colonization of colon cancer, and its inhibition may represent a novel approach for limiting cancer progression. Recent studies have also shown that arginase activity, including that of both ARG1 and ARG2, can activate the Akt and mTOR signaling pathways in various cell types [41, 42]. As the PI3K/Akt/mTOR pathway plays a pivotal role in regulating cell proliferation, apoptosis, angiogenesis, and metastasis, its dysregulation contributes to cancer development and therapeutic resistance. Consistent with these findings, we observed that ARG1 overexpression activates phosphorylation of the PI3K/Akt/mTOR pathway in CRC cells. Importantly, our study demonstrated that LA simultaneously exerted antitumor effects by targeting ARG1 and directly inhibiting PI3K/Akt/mTOR signaling in both CRC cells and patient-derived organoids. In addition, LA significantly increased the proportion of CD8⁺ T cells while reducing the expression of CD69⁺ and PD-1⁺ cells in the TIME, indicating an alleviation of T cell exhaustion. Collectively, these results highlight the dual role of LA in modulating both tumor cell signaling and immune responses, offering new insight into potential strategies for CRC treatment.

Proteomics and metabolomics are powerful tools for deciphering the multi-target regulatory mechanisms of TCM. Unlike conventional therapies that often act through single-target mechanisms, TCM exerts its antitumor effects through the coordinated modulation of multiple biological processes, including tumor cell proliferation, apoptosis, invasion, and immune regulation within the tumor microenvironment. The integration of proteomic and metabolomic analyses enables the construction of a multidimensional "drug–protein–metabolite" interaction network and provides molecular-level evidence to support the pharmacological basis and mechanisms of TCM. In our study, the proteomic and metabolomic data revealed an enrichment of arginine metabolism-related pathways upon LA treatment. This observation led us to hypothesize that LA may exert its antitumor effects through modulation of arginine metabolism. Arginine metabolism primarily follows two major pathways: one catalyzed by ARG1/2 to produce ornithine and urea, and the other catalyzed by NOS to generate citrulline and nitric oxide. Among the downstream products of these pathways, polyamines represent a key group of metabolites with established roles in tumor progression [43]. Our results showed that the expression levels of ARG1, ARG2, and NOS2 were all reduced in the combination treatment group. However, the difference in NOS2 expression between the Oxa and LA + Oxa groups was not statistically significant, indicating that the NOS2-dependent pathway may not be the principal mechanism through which LA exerts its distinct effects. Therefore, we focused subsequent mechanism investigations on the ARG1-mediated metabolic pathway.

Organoid models offer a highly biomimetic experimental platform for investigating the antitumor mechanisms of TCM. Conventional two-dimensional (2D) cell culture systems fail to replicate the three-dimensional architecture and cellular heterogeneity of tumor tissues, while animal models are constrained by interspecies differences, high costs, and lengthy experimental timelines. In contrast, organoids derived from patient tumor tissues faithfully preserve the histoarchitecture, cellular composition, and genetic features of the original tumor and can be stably propagated over long periods [44]. These advantages make organoids particularly valuable in TCM research. In this study, we employed three types of patient-derived CRC organoid models: 3D cultured organoids, organoid–lymphocyte co-culture systems, and ALI organoids. The 3D cultured organoids, primarily derived from tumor stem cells, are commonly used to evaluate the direct cytotoxic effects of therapeutic agents. In contrast, both the organoid–lymphocyte co-culture system and the ALI organoid model recapitulate key aspects of the TIME in vitro and are suitable for investigating drug-induced immunomodulatory effects. Our results support the feasibility and complementarity of these three models in assessing both the direct antitumor effects of LA and its regulatory impact on TIME. This multi-model approach provides a valuable methodological reference for future studies on the mechanisms of TCM in cancer treatment.

While this study provides insights into the efficacy and underlying mechanisms of the LA-Oxa combination in CRC treatment, several important questions remain to be addressed in future research. First, we demonstrate that LA modulates ARG1 expression within the TIME to alter T cell exhaustion-related features, but the precise molecular mechanisms underlying this regulation warrant further investigation. Second, due to time and resource limitations, ARG1-knockout or knockdown tumor models could not be established in vivo. Future studies employing such models will be necessary to further substantiate the mechanistic role of ARG1. Despite these limitations, the convergence of our in vitro genetic, in vivo pharmacological, and multi-omics data provides a coherent and robust foundation for our conclusions. Third, although our findings confirm that LA enhances the antitumor effects of Oxa, the optimal dosage ratio and scheduling of this combination therapy should be systematically evaluated to maximize its therapeutic potential. Additionally, although ARG2 may contribute to arginine metabolism in certain contexts, current evidence does not strongly support its central involvement in LA-mediated modulation in CRC. Nevertheless, the functional role of ARG2 in CRC progression and its potential contribution to the combined effects observed in this study warrant further investigation.

## Conclusion

Our study showed that LA enhances the antitumor efficacy of oxaliplatin in CRC both in vitro and in vivo. We further show that LA is associated with changes in ARG1 expression and arginine metabolism, along with reduced CRC cell proliferation and alterations in T cell exhaustion-related features within the TIME. These findings offer new insights into the pharmacological regulation of ARG1 and suggest that combining LA with standard chemotherapy may represent a promising strategy to overcome drug resistance and improve clinical outcomes in CRC patients.

## Data Availability

Data generated and/or analyzed during the current study are available from the corresponding author on reasonable request.

## Abbreviations

CRC: Colorectal cancer
Oxa: Oxaliplatin
LA: Licoisoflavone A
ARG1/2: Arginase-1/2
NOS2: Nitric oxide synthase 2
PBMC: Peripheral blood mononuclear cell
nor-NOHA: Nω-hydroxy-nor-arginine
CCK-8: Cell Counting Kit-8
ALI: Air–liquid interface
CTG: CellTiter-Glo^®^
FASP: Filter-aided sample preparation
ALT: Alanine aminotransferase
AST: Aspartate aminotransferase
SPR: Surface plasmon resonance
PI3K: Phosphoinositide 3-kinase
Akt: Protein kinase B
mTOR: Mammalian target of rapamycin
TIME: Tumor immune microenvironment
